# Exposure to Maternal Gestational Diabetes Is Associated With Higher Cardiovascular Responses to Stress in Adolescent Indians

**DOI:** 10.1210/jc.2014-3239

**Published:** 2014-12-05

**Authors:** Ghattu V. Krishnaveni, Sargoor R. Veena, Alexander Jones, Krishnamachari Srinivasan, Clive Osmond, Samuel C. Karat, Anura V. Kurpad, Caroline H. D. Fall

**Affiliations:** Epidemiology Research Unit (G.V.K., S.R.V., S.C.K.), CSI Holdsworth Memorial Hospital, Mysore 570021, India; Centre for Cardiovascular Imaging (A.J.), University College London Institute of Cardiovascular Science, London W1T 7HA, United Kingdom; St John's Research Institute (G.V.K., K.S., A.V.K.), St John's National Academy of Health Sciences, Bangalore 560034, India; and Medical Research Council Lifecourse Epidemiology Unit (C.O., C.H.D.F.), University of Southampton, Southampton SO16 6YD, United Kingdom

## Abstract

**Context:**

Altered endocrinal and autonomic nervous system responses to stress may link impaired intra-uterine growth with later cardiovascular disease.

**Objective:**

To test the hypothesis that offspring of gestational diabetic mothers (OGDM) have high cortisol and cardiosympathetic responses during the Trier Social Stress Test for Children (TSST-C).

**Design:**

Adolescents from a birth cohort in India (n = 213; mean age, 13.5 y), including 26 OGDM, 22 offspring of diabetic fathers (ODF), and 165 offspring of nondiabetic parents (controls) completed 5 minutes each of public speaking and mental arithmetic tasks in front of two unfamiliar “evaluators” (TSST-C). Salivary cortisol concentrations were measured at baseline and at regular intervals after the TSST-C. Heart rate, blood pressure (BP), stroke volume, cardiac output, and total peripheral resistance were measured continuously at baseline, during the TSST-C, and for 10 minutes after the test using a finger cuff; the beat-to-beat values were averaged for these periods.

**Results:**

Cortisol and cardiosympathetic parameters increased from baseline during stress (*P* < .001). OGDM had greater systolic BP (mean difference, 5.6 mm Hg), cardiac output (0.5 L/min), and stroke volume (4.0 mL) increases and a lower total peripheral resistance rise (125 dyn · s/cm^5^) than controls during stress. ODF had greater systolic BP responses than controls (difference, 4.1 mm Hg); there was no difference in other cardiosympathetic parameters. Cortisol responses were similar in all three groups.

**Conclusions:**

Maternal diabetes during pregnancy is associated with higher cardiosympathetic stress responses in the offspring, which may contribute to their higher cardiovascular disease risk. Further research may confirm stress-response programming as a predictor of cardiovascular risk in OGDM.

Dysregulation of the hypothalamic-pituitary-adrenal (HPA) axis leading to increased cortisol concentrations and altered cardiac sympathetic reactivity to stress have been linked to higher metabolic and cardiovascular disease risk in humans (1). Fetal exposure to maternal undernutrition and maternal stress or excess of glucocorticoids may permanently alter cortisol and cardiosympathetic stress-responsiveness, leading to a range of metabolic and cardiovascular changes in the offspring (2). This may contribute to the higher risk of type 2 diabetes and cardiovascular disease in low-birth-weight individuals (1). Exposure to maternal overnutrition, as in gestational diabetes mellitus (GDM), also increases metabolic and cardiovascular disease risk in the offspring (3). Animal model studies show that these offspring may be at an increased risk of hypertension and cardiovascular abnormalities due to altered vascular reactivity and endothelial dysfunction (4). Limited studies in animals have indicated that higher sympathetic nervous system activity also leads to hypertension in offspring of diabetic mothers (5, 6). However, the role of stress-response programming as a cause of higher cardiovascular disease risk in offspring exposed to intrauterine hyperglycemia has not been studied in humans.

The Mysore Parthenon birth cohort study is at the forefront of investigating the long-term effects of maternal GDM on the offspring in India (7, 8). We showed earlier in this cohort that maternal, but not paternal, diabetes was strongly associated with higher cardiovascular risk markers in the offspring during childhood (8). Even in control children, maternal insulin concentrations were associated positively with offspring subscapular skinfold thickness and homeostasis model assessment for insulin resistance (HOMA-IR). We have now re-examined the cohort to test whether offspring of gestational diabetic mothers (OGDM) exhibit exaggerated cortisol and cardiosympathetic responses to a laboratory-induced stressor compared with control offspring and whether similar stress responses are observed in offspring of diabetic fathers (ODF). Availability of continuous measures of glucose and insulin concentrations in the mothers also enabled us to test whether glucose and insulin concentrations in the absence of diabetes are associated with stress responses in the offspring.

## Subjects and Methods

During 1997–1998, we screened 1539 women booking consecutively into the antenatal clinics of Holdsworth Memorial Hospital (HMH). Of these, 1233 (80%) eligible women (<32 week gestation at booking, no diabetes before pregnancy, planned delivery at HMH, singleton pregnancy) were invited to undergo detailed anthropometry and a 100-g, 3-hour, oral glucose tolerance test (7). Of the 830 women (67%) who participated, 785 completed the oral glucose tolerance test (49 with GDM). A total of 639 women with known GDM status (81%) delivered at HMH (43 OGDM); six babies were stillborn, and three were born with severe congenital anomalies. The remaining 630 offspring were included for further follow-up (41 OGDM). The babies were measured at birth and at 6- to 12-monthly postnatal follow-ups. Paternal diabetes status was assessed using fasting glucose at the 5-year follow-up. Offspring of non-GDM mothers and nondiabetic fathers were designated controls.

At 13.5 years, 445 adolescent children were available for follow-up. We aimed to perform a standard laboratory-based stress test, the Trier Social Stress Test for Children (TSST-C) (9), in 200 offspring of non-GDM mothers, representing four birth weight categories, and all available OGDM (n = 32) from those living within Mysore city (n = 298). Families were approached in the chronological order of the children's dates of birth until the final number was achieved (234; 28 OGDM, 23 ODF).

### Trier Social Stress Test for Children

The tests were conducted between 2 and 3:30 pm, for one child at a time (10). A baseline (pretest) salivary sample was collected 10 minutes before the test, after subjects had watched a calming video for 5 minutes in a standing position. The children performed 5 minutes of public speaking (imaginative storytelling) and 5 minutes of mental arithmetic standing in front of two unfamiliar adults who pretended to evaluate their performance. Further salivary samples were collected at 0, 10, 20, 30, and 60 minutes after the TSST-C to measure the cortisol response. Children watched another calming video for 5 minutes, in the standing position, before the final salivary sample was collected.

Systolic and diastolic blood pressure (BP), cardiac output, stroke volume, heart rate, and total peripheral resistance (TPR) were measured continuously during the pretest video-viewing period (baseline), during the TSST-C, and for 10 minutes after the TSST-C. This was done using a noninvasive, portable hemodynamic monitoring system with appropriately sized finger cuffs (Nexfin; BMEYE). The beat-to-beat values were averaged over 5 minutes for the baseline, story, mental arithmetic, and immediate poststressor periods.

Detailed anthropometry was performed. Bioimpedance-estimated percentage body fat (fat%) and resting BP were measured as described before (8). A fasting blood sample was collected the following day for measuring plasma glucose, insulin, and lipid concentrations. Pubertal growth was assessed as the stage of breast development in girls and genital development in boys using Tanner's method (11). The socioeconomic status of the family was determined using the Standard of Living Index designed by the National Family Health Survey-2 (12). Physical activity was measured using Actigraph accelerometers (AM7164/GT1M; MTI; Health Services) which measure movement in the vertical plane as counts. Children's energy, fat, and protein intakes were measured using a purpose-designed food frequency questionnaire at 9.5 years of age.

Laboratory assays were carried out at the Diabetes Unit, KEM Hospital Research Center, Pune, India. Salivary cortisol concentrations were measured using an ELISA method (Alpco Diagnostics) according to the manufacturer's instructions. The assay sensitivity was 1 ng/mL; inter- and intra-assay coefficients of variation were 10.0 and 6.6%, respectively.

Plasma glucose, insulin, and fasting lipid concentrations were measured as described elsewhere (8, 13). Insulin resistance was estimated using the HOMA-IR equation (14) because this was the method used in previous follow-ups. Reassuringly, there were high correlations between these values and HOMA-IR calculated using the online HOMA calculator (r = 0.99) (15). Area-under-the-curve measures were calculated using the trapezoid rule for maternal plasma glucose (GAUC) and insulin (IAUC) (16).

The TSST-C judges and the laboratory staff were not aware of parental diabetes status.

The HMH ethics committee gave approval for the study; informed written consent from parents and assent from children were obtained.

### Statistical methods

Children's salivary cortisol and insulin concentrations and HOMA-IR distributions were skewed and were log-transformed for analysis. Differences between OGDM and controls and between ODF and controls in anthropometry, risk factors, and cortisol and cardiosympathetic parameters at baseline were analyzed using independent *t* tests or χ^2^ tests. The above differences were adjusted for age, sex, socioeconomic status, and children's current weight by including these variables as covariates in multiple linear or logistic regression models. We performed linear mixed-model analysis to examine the associations of maternal GDM and paternal diabetes with repeated measures of outcome variables to account for within-group correlations. Salivary cortisol concentrations at all timepoints and averaged cardiosympathetic parameters at different test stages, respectively, were included in the models to examine the change from baseline over time, adjusted for age, sex, and socioeconomic status. The cardiac parameters are all related to body size. Traditionally, cardiac output, stroke volume, and TPR are indexed on body surface area. We explored which body size parameters (weight, height, higher-order terms of these) best explained the variability in the above cardiovascular outcomes. Body weight alone was the best, and this was also adjusted for in all regressions by including this variable as a covariate. Analyses were also repeated using indexed variables, and after adjusting for other measures of body size such as body mass index (BMI) and fat% instead of weight. Analyses were done using SPSS version 21 (IBM Corp) and STATA version 12 (StataCorp).

## Results

Of the 234 participants, 231 completed the TSST-C. Adequate pre- and post-test salivary samples and complete cardiosympathetic responses were available for 213 children, including 26 OGDM, 22 ODF, and 165 controls. Birth weight, current BMI, and socioeconomic status did not differ significantly between participants and nonparticipants.

**Figure 1.**
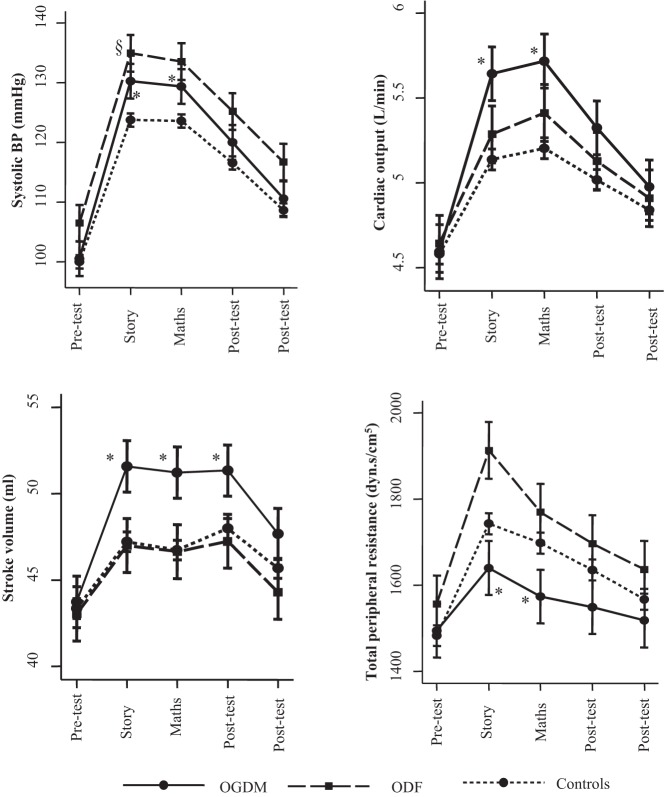
Stress-induced cardiosympathetic response in OGDM, ODF, and controls. Values represent mean (95% CI) in OGDM, ODF, and controls at different timepoints relative to the stress test. Values were derived using linear mixed-model analysis, adjusting for age, sex, socioeconomic status, and current weight. *, *P* < .05 for the difference between OGDM and controls; §, *P* < .05 for the difference between ODF and controls.

In all children, the TSST-C resulted in significant cortisol (Supplemental Figure 1) and cardiosympathetic responses (Figure 1), indicated by a significant effect of time in mixed-model analysis (*P* < .001 for all). There was a high degree of correlation between mean cardiosympathetic parameters during the story and mental arithmetic tasks (r > 0.9 for all except TPR; r = 0.8 for TPR).

### Offspring of gestational diabetic mothers

General characteristics of OGDM in comparison to control offspring at 13.5 years of age are presented in Table 1. OGDM were heavier and more adipose and had a higher prevalence of overweight/obesity and higher fasting insulin and insulin resistance (HOMA-IR) than control children, even after controlling for age, sex, and socioeconomic status in multiple regression analyses. In both sexes, OGDM had similar pubertal stages to control children. There were no differences in resting BP or fasting glucose and lipid concentrations between the two groups. They were also similar to control children in physical activity counts.

**Table 1.** Anthropometry, Cardiometabolic Risk Factors, and Baseline Cortisol Concentrations and Cardiosympathetic Parameters in OGDM, Controls, and ODF Who Participated in the TSST-C at Age 13.5 y

	OGDM	P1	Controls	P2	ODF
n	26		165		22
Age, y^a^	13.5 (0.1)	.8	13.6 (0.1)	.2	13.5 (0.1)
Boys/girls, n (%)^a^	8/18 (31/69%)	.03	89/76 (54/46%)	.7	11/11 (50/50%)
BMI, kg/m^2^	20.1 (3.4)	<.001	17.4 (2.6)	.6	17.5 (2.5)
Height, cm	154.9 (7.6)	.5	154.1 (7.4)	.8	154.9 (7.0)
Subscapular skinfolds, mm	17.4 (14.3, 25.3)	<.001	12.1 (8.2, 15.8)	.4	9.8 (8.1, 20.0)
Fat%	26.4 (8.0)	.02	21.3 (7.5)	.8	22.1 (7.6)
Overweight/obesity, n (%)	8 (30.8%)	.008	15 (9.1%)	.5	1 (4.5%)
Physical activity, counts/min	447.5 (150.6)	.6	470.3 (176.4)	.1	413.3 (124.7)
Energy intake, kcal/d	2426 (658)	.7	2561 (844)	.7	2565 (1150)
Fat intake, g/d	77 (30)	.8	82 (33)	.8	77 (41)
Protein intake, g/d	58 (18)	.9	59 (20)	.6	60 (26)
Socioeconomic status, score^a^	38.5 (6.7)	1.0	38.6 (6.7)	.6	39.3 (5.9)
Maternal GAUC, mmol	1708 (545)	<.001	1121 (149)	.8	1115 (167)
Maternal IAUC, pmol	80 404 (35 782)	.001	58 018 (30 633)	1.0	59 230 (40 832)
Resting parameters					
Systolic BP, mm Hg^b^	110.5 (8.1)	.1	109.0 (8.3)	.1	111.5 (8.0)
Diastolic BP, mm Hg^b^	61.7 (6.5)	.3	61.4 (7.0)	.4	62.3 (7.0)
Triglycerides, mmol/L	0.83 (0.4)	.9	0.82 (0.4)	.3	0.91 (0.4)
Total cholesterol, mmol/L	3.7 (0.7)	.3	3.6 (0.6)	.3	3.4 (0.6)
HDL cholesterol, mmol/L	1.08 (0.2)	.7	1.10 (0.2)	.3	1.03 (0.3)
Glucose, mmol/L	5.1 (0.4)	.7	5.1 (0.5)	.6	5.0 (0.5)
Insulin, pmol/L	54.3 (37.0, 73.3)	.02	42.5 (30.7, 53.2)	.09	49.6 (38.9, 63.5)
Insulin resistance (HOMA-IR)	2.0 (1.5, 2.6)	.02	1.6 (1.1, 2.0)	.1	1.8 (1.5, 2.2)
TSST-C parameters					
Cortisol concentrations, ng/mL	5.9 (5.2, 8.3)	.6	6.6 (4.6, 9.1)	.2	7.9 (7.1, 9.0)
Systolic BP, mm Hg^c^	101.2 (9.3)	.6	100.1 (11.9)	.02	106.3 (12.1)
Diastolic BP, mm Hg^c^	68.6 (5.9)	.9	69.1 (8.0)	.003	74.3 (7.6)
Heart rate, bpm	106.7 (13.9)	.7	107.1 (12.1)	.4	109.5 (13.0)
Cardiac output, L/min^d^	4.8 (0.9)	.8	4.6 (0.8)	.8	4.6 (0.7)
Stroke volume, mL^d^	45.6 (8.1)	.7	43.1 (7.9)	.8	42.9 (7.8)
TPR, dyn·s/cm^5^^d^	1414 (208)	.8	1494 (305)	.1	1565 (220)

Abbreviation: HDL, high-density lipoprotein. Values are expressed as mean (SD), median (IQR), or number (percentage). P1 is for the difference between OGDM and controls; P2 is for the difference between ODF and controls.

a Unadjusted *P* values.

b Resting BP, measured using automated BP machine at the arm level in sitting position.

c TSST-C BP, measured over 5 minutes using finger cuff, in standing position.

d Current weight.

#### Trier Social Stress Test for Children

At baseline, there were no differences between OGDM and control children in salivary cortisol concentrations and cardiosympathetic parameters (Table 1). There was no significant association between maternal GDM and cortisol responses to TSST-C (Supplemental Figure 1). Mixed-model analysis showed that in OGDM, there was an increase in systolic BP from baseline by about 5–6 mm Hg more than that in controls, after adjusting for age, sex, socioeconomic status, and current weight (Table 2 and Figure 1). Similarly, there was a significantly greater increase in cardiac output and stroke volume, but a lesser increase in TPR in OGDM compared to controls during the TSST-C (Figure 1 and Table 2). These associations were similar during public speaking and mental arithmetic tasks (Table 2) and remained statistically significant after adjustment for current BMI or fat% instead of weight, and after additionally adjusting for activity and dietary intake (data not shown). The findings were also robust to further adjustment of cardiac output (*P* < .001), stroke volume (*P* = .001), and TPR (*P* = .02) for body surface area. The associations were similar in boys and girls, and there were no interactions with sex for these outcomes after complete adjustment (*P* ≥ .2).

**Table 2.** Association Between Parents' Diabetes Status and Offspring Cardiovascular Responses to Stress

	Public Speaking				Mental Arithmetic			
	Systolic BP, mm Hg	Cardiac Output, L/min	Stroke Volume, mL	TPR, dyn·s/cm^5^	Systolic BP, mm Hg	Cardiac Output, L/min	Stroke Volume, mL	TPR, dyn·s/cm^5^
Maternal GDM								
Model 1								
β (95% CI)	5.96 (2.10, 9.82)	0.49 (0.26, 0.72)	3.98 (2.00, 5.97)	−114 (−220, −9)	5.23 (1.37, 9.10)	0.50 (0.27, 0.73)	4.10 (2.12, 6.09)	−136 (−241, −30)
*P*	.002	<.001	<.001	.03	.008	<.001	<.001	.01
Model 2								
β (95% CI)	5.96 (2.10, 9.82)	0.49 (0.26, 0.72)	3.98 (2.00, 5.97)	−114 (−220, −9)	5.23 (1.37, 9.10)	0.50 (0.27, 0.73)	4.10 (2.12, 6.09)	−136 (−241, −30)
*P*	.002	<.001	<.001	.03	.008	<.001	<.001	.01
Paternal diabetes								
Model 1								
β (95% CI)	4.70 (0.54, 8.85)	0.09 (−0.16, 0.34)	0.09 (−2.05, 2.22)	97 (−17, 211)	3.47 (−0.68, 7.62)	0.15 (−0.10, 0.40)	0.22 (−1.92, 2.35)	−1 (−115, 112)
*P*	.03	.5	.9	.09	.1	.2	.8	1.0
Model 2								
β (95% CI)	4.70 (0.54, 8.84)	0.09 (−0.16, 0.34)	0.09 (−2.05, 2.22)	97 (−17, 211)	3.47 (−0.68, 7.62)	0.15 (−0.10, 0.40)	0.22 (−1.92, 2.35)	−1 (−115, 112)
*P*	.03	.5	.9	.09	.1	.2	.8	1.0

The beat-to-beat outcome values were averaged over 5 minutes for baseline, story, mental arithmetic, and immediate post-stressor periods for analysis; β and *P* values were derived using linear mixed-model analysis, with offspring outcomes as the continuous variable and parental diabetes status as “yes/no” categories. β represents unit change in the outcome variable in the OGDM/ODF from baseline in excess of that in controls. Model 1 was adjusted for children's age and sex; model 2 represents model 1 + socioeconomic status and children's current weight.

### Offspring of diabetic fathers

At 13.5 years, after adjustment for age, sex, and socioeconomic status, ODF tended to have higher HOMA-IR than control children (Table 1). Pubertal stage in male and female ODF was similar to control boys and girls, respectively. There were no differences between ODF and controls in anthropometry and cardiovascular risk markers.

#### Trier Social Stress Test for Children

Pretest systolic BP was significantly higher in ODF than control offspring (Table 1). Mixed-model analysis showed that there was also a significant increment in systolic BP from baseline during TSST-C compared to controls (Figure 1); the association was little changed after adjustment for age, sex, socioeconomic status, and current weight (Table 2). There were no associations between paternal diabetes status and cortisol (Supplemental Figure 1) or other cardiosympathetic responses to stress (Table 2).

### Control children

Even in the control offspring, higher maternal insulin concentrations (IAUC) were associated with higher responses for diastolic BP (0.0001 mm Hg per pmol · h/L change in maternal IAUC; 95% confidence interval [CI], 0.00002–0.0001 mm Hg; *P* = .02) and TPR during the TSST-C (0.002 dyn · s/cm^5^ per pmol · h/L change in maternal IAUC; 95% CI, 0.001–0.003 dyn · s/cm^5^; *P* < .01, adjusted). Maternal IAUC was not associated with cortisol and other cardiosympathetic responses to stress. There was no association between maternal glucose concentrations (GAUC) and stress responses in the control offspring (data not shown).

## Discussion

In a sample of healthy adolescent children from a birth cohort in India, OGDM exhibited greater cardiosympathetic responses to a standard stress test than control offspring. ODF also had greater stress-induced systolic BP compared to controls; there were no apparent differences from controls in the other stress-response parameters. Cortisol responses to stress were similar in all three groups. As far as we know, this is the first study to report neuroendocrine stress-responsiveness in relation to parental diabetes status. As observed during the previous follow-ups of the same cohort, OGDM and ODF had higher insulin resistance (HOMA-IR) compared to control children.

Our study has demonstrated the first evidence of exaggerated cardiosympathetic stress responses in OGDM. It has been proposed that altered HPA axis and autonomic nervous system responses to psychological stress increase susceptibility to cardiovascular disease (1). Using a well-validated stress test, we showed that OGDM develop higher systolic BP, cardiac output, and stroke volume than control children under stressful situations. These responses were independent of their body size, body surface area, or fat%. TPR response to stress was lower than in control children; this may be due to the reciprocal actions of cardiac output and TPR in the regulation of BP, especially in stress, in which an increase in one of these parameters is compensated for by a decrease in the other (17). Although there was also a greater stress response in ODF compared to controls, suggesting gene interactions, it was not as strong as in OGDM and was apparent only with systolic BP. However, we cannot rule out stress responses in other cardiovascular parameters such as arterial compliance in ODF, which our study did not measure. There was also some evidence of higher cardiosympathetic stress responses in association with higher maternal insulin concentrations, even in the absence of parental diabetes. However, our study does not provide conclusive evidence for altered stress responses in relation to variations in maternal glycemia within the normal range in control offspring.

Studies show that OGDM are at an increased risk of obesity, type 2 diabetes, and cardiovascular disease in later life (3, 18–20). The Mysore Parthenon study had previously observed higher sc adiposity, insulin resistance (HOMA-IR), and systolic BP in OGDM during childhood, more strongly than ODF, compared to control offspring (8). Our current investigations show that these children continue to have higher metabolic risk markers even during early adolescence. The mechanism underlying higher cardiovascular and metabolic risks in OGDM is not well understood. Although genetic transmission may offer a partial explanation, studies among Pima Indians, showing a higher risk of obesity and type 2 diabetes in OGDM than ODF (18), and in siblings born after the mother became diabetic compared to those born before (21) suggest an incremental role of intrauterine hyperglycemia. Intrauterine hyperinsulinemia, resulting from increased glucose transfer from the mother, is thought to play a major role in promoting long-term adverse outcomes. Apart from the anabolic properties of insulin, contributing to increased size, hyperinsulinemia and/or leptin resistance in OGDM may also alter hypothalamic appetite regulation, resulting in increased food intake and obesity (22). Shared maternal lifestyle habits, such as higher energy intake, may also be important (23). Dysfunctional endothelial systems leading to impaired vascular dilatation may result in hypertension and cardiovascular abnormalities (4). Our current findings indicate that altered physiological stress responses may also be a contributing factor.

A few studies have shown an association between low birth weight, a proxy for impaired fetal growth, and higher cortisol (24, 25) and cardiosympathetic stress responses (26, 27), suggesting a mechanistic link between early growth and later cardiovascular disease in humans. Although maternal GDM also influences fetal growth adversely, studies examining stress-responsiveness in OGDM are sparse. In an earlier study from the United Kingdom, 24-hour urinary glucocorticoid metabolite excretion was higher in both low- and high-birth-weight children (28). The researchers speculated that the latter association may be due to altered HPA axis activity in babies born to mothers with glucose intolerance during pregnancy. Studies exploring autonomic stress responses in OGDM are also very few. Limited data from animal studies suggest altered sympathetic activity in OGDM (5, 6). In a rat model, pups born to mothers with streptozotocin-induced diabetes had higher renal sympathetic activity and developed hypertension later in life (5). In another study, norepinephrine levels were increased, at 22 and 64 days postnatally, in the heart tissue of rat pups born to diabetic mothers (6). These studies, however, did not explore the effect of stress on sympathetic activity. Our study significantly extends this limited literature on the role of neuroendocrine development in the early origins of adult chronic disease.

We do not know the mechanism for altered cardiosympathetic stress responses in our OGDM population. This may be related to a direct effect of glucose and insulin concentrations on the autonomic nervous system. Although cardiovascular autonomic dysfunction is a known complication of uncontrolled hyperglycemia in diabetic individuals (29), this is unlikely in our healthy children born to GDM mothers, whose glucose concentrations were within the normal range and were not different from that of the control children. Hyperinsulinemia, which is a feature in the OGDM in our cohort, may also increase their sympathetic activity (30); however, cardiosympathetic parameters were similar in OGDM and controls in an unstressed state in our study. Another possible explanation is that perinatal hyperinsulinemia in OGDM results in altered neurobehavioral development (31). An animal model study showed that rat pups born to diabetic mothers demonstrated greater anxiety in stressful situations (32). Diabetic model studies in animals show that maternal hyperglycemia and/or hyperinsulinemia during fetal development permanently alter the development of the hypothalamic structure and function, including that of the paraventricular nucleus, which regulates anterior pituitary and autonomic nervous system functions (31). It is suggested that intrauterine hyperglycemia induces oxidative stress in the developing brain cells leading to cell inflammation, which may underlie the hypothalamic developmental aberrations in OGDM (31, 33). Alternatively, in our cohort, OGDM had higher cognitive ability than controls at 9–10 years of age (34), which may have resulted in altered stress perception. Interestingly, OGDM did not show altered cortisol responsiveness. This may be related to inadequate power due to our small GDM group. Cortisol response to stress has shown to be gender specific and apparent only in male offspring in studies examining birth weight associations of stress (24). It is possible that it is also true in OGDM, and because we have very few boys in the OGDM group, the cortisol responses may not be apparent. However, it is beyond the scope of our study to determine the mechanism for our findings.

### Strengths and weaknesses

This is the first study to use a well-established stress test to compare stress responses of OGDM and ODF with controls in humans. Continuous measures of maternal glucose and insulin concentrations were examined in association with the above responses even in control children. This is also one of the few prospective non-Pima Indian studies to examine the long-term risks of intrauterine exposure to diabetes in comparison with paternal diabetes in children. A limitation was the relatively small numbers of OGDM. Another limitation was that the TSST-C was conducted only in those children living within the Mysore city. Because cognitive performance was better in urban children compared to rural children in our cohort (34), their orientation toward stressful situations may have differed, too. However, because most of our OGDM were from an urban background, selection bias is unlikely. The fathers' diabetes status was not measured during the index pregnancy and was determined using only fasting glucose concentrations. In contrast to OGDM, ODF were not overselected. These factors may have reduced the power of any association between paternal diabetes and offspring stress response.

In conclusion, our findings indicate that OGDM are not only exposed to higher adiposity and insulin resistance but are also susceptible to higher stress reactivity. Although it is not known whether the higher cardiovascular reactivity in relation to a laboratory stressor in OGDM predicts future disease risk, a recent meta-analysis showed that greater reactivity to laboratory-induced stress was associated with an increased risk of future negative cardiovascular outcomes (35). Because individuals who have greater reactivity to a laboratory stressor are also more likely to have higher cardiovascular reactivity to everyday stressors (35), these differences in OGDM from normal controls may combine to increase the long-term risk of disease substantially. More studies in OGDMs from different populations are required to substantiate the findings from our study.
